# Introducing the Muzzammil classification for spoke wheel injuries in children to enhance injury assessment and treatment in developing countries

**DOI:** 10.1038/s41598-023-46255-0

**Published:** 2023-11-07

**Authors:** Muhammad Muzzammil, Muhammad Saeed Minhas, Uzair Yaqoob, Syed Ghulam Mujtaba Shah, Syed Jahanzeb, Abdul Qadir, Sohail Ahmed Fazlani, Saadia Jabbar

**Affiliations:** 1 Department of Orthopedics, Sindh Gov. Services Hospital, Karachi, Pakistan; 2 Jinnah Postgraduate Medical Center, Karachi, Pakistan; 3 Surgical Department, Dr Ruth K M Pfau Civil Hospital, Karachi, Pakistan; 4 Department of Orthopedics, Dr Ruth K M Pfau Civil Hospital, Karachi, Pakistan; 5 Department of Surgery, Jinnah Postgraduate Medical Center, Karachi, Pakistan

**Keywords:** Health care, Medical research

## Abstract

Motorbike spoke wheel injuries (SWIs) among children are a notable public health concern, especially in low and middle-income regions. The primary objective of this study is to comprehensively examine the patterns of motorbike spoke wheel injuries (SWIs) in children. Additionally, the study introduces a novel classification system for these injuries. The implementation of this classification system aims to streamline the management of SWIs, making it more efficient and facilitating the development of standardized treatment protocols. This prospective observational study was conducted in the Accident and Emergency Department from January 2019 to 2021. Children < 14 years of age of either gender with foot and ankle injury due to motorbike spoke wheels as passengers and presenting within 3 days were included. The motorbike SWI was assessed for its location and classified by a new classification as Class I, Soft tissue injury without skin loss; Class II, Skin loss of more than 1 cm without underlying tissue involvement; Class III, Skin loss with underlying tissue involvement, this class is further divided on basis of underlying soft-tissue involvement; Class IV: mangled foot/toe. Management plan and outcome were noted. In our study158 children suffering from SWI were registered with a mean age of 6.2 ± 5.4 years, 127 (80.37%) males and 31 (19.62%) females. Class I injury was seen in 18 (11.39%) patients, class II in 69 (43.67%), and class III in 68 (43.03%) patients. Class III injuries were further subcategorized as follows: IIIT (Tendon) injuries, which accounted for 32 cases (20.25%); IIIB (Bone) injuries, with 29 cases (18.35%); and IIINV (Neurovascular) injuries, identified in 7 cases (4.43%). Class IV injuries were observed in 3 (1.8%) children. The flap was needed in 33 (20.88%) patients. There were no complications like flap necrosis or graft rejection. In this current study, a new classification system specific for a motorbike SWI has been introduced along with its application on children presenting at tertiary care hospital’s emergency department. The application of the proposed classification will enable universal management guidelines for SWIs, especially in the Ino-Pak region where SWIs are common.

## Introduction

Motorbike injuries are becoming very common when road traffic accidents are considered in the urban and suburban areas of developing countries like Pakistan^1–5^. Motorbike spoke wheel injuries (SWIs) are a significant category of injuries primarily affecting children and represent a growing public health concern, especially in regions with a high prevalence of motorbike usage. Motorbike Spoke wheel injuries (SWIs) occur when the foot of the passenger is trapped in the rotating spokes of the wheel of a motorbike^6^. In 1948, Riess documented bicycle spoke wheel injuries, and in 1978, Ahmad described motorbike spoke wheel injuries^7,8^. Compared with the bicycles, the motorbike caused more severe SWIs injuries due to their higher energy^7,9–11^. Most of the inhabitants of Karachi belong to the lower middle class and rely on motorbikes as a means of transportation to drop their children to school as they cannot afford school bus services or private cars for transportation. It is becoming highly prevalent, especially in our part of the world because of the lack of safety protocols. Children are the main victims^12^. SWI can range from a minor soft tissue laceration to an extensive crush injury involving tendons, and bones, ultimately leading to subtotal amputations, mainly involving the heel and ankle^12–14^. Children of 5–10 years old are more prone to motorbike SWIs. A prevalence rate of 10.3% had been reported in Nigeria^14^, and an incidence rate of 21.7% has been calculated in Lahore, Pakistan^15^. Children’s feet are often dangling near the wheels because they cannot reach the footrest of the motorbike due to their smaller size and are thus prone to entrapment in the spoke wheels^14^. The occurrence of SWI is exacerbated by multiple passengers on a single motorbike and bumpy roads^16^.

In this study, we include only motorbike-related SWIs. Motorbikes having higher mass and velocity are more likely to produce a greater impact (high energy) on the foot when it is caught in the spoke wheel. Moreover, a bicycle rider immediately stops the bicycle when the injury occurs, but the motorbike has to be switched off following the injury and the foot is trapped between the spokes and horizontal bar and severely injured. The severity of injury also depends upon the distance of the tissue injured from the center of the wheel and also on the rate of deceleration of the vehicle after the impact, which are difficult parameters to assess.

The most susceptible part, of this type of injury, is the heel and the treatment is very challenging because of the complex nature of the injury usually involving skin, tendons, and bones^16^. Management of the SWI is often challenging and is based on the location and grade of injury. Although there is no universally accepted classification for motorbike SWIs, Tscherne and Oestern classification is preferred by many authors^17,18^. In this classification, injuries are graded as closed soft tissue injury with contusion (grade 0), minor bruises, and lacerations (grade I), major soft tissue loss (grade II), tendon rupture, and neurovascular injury, and fractures (grade III).

Management of SWI may involve multiple surgeries, from debridement of the dead and devitalized tissues to the repair of tendons, and fixation of fractures, grafts, and flaps to salvage the limbs^13,14^. We proposed a new classification to simplify SWI and its management plan, implementing this classification for patients coming to the emergency department with a history of motorbike SWI. Our study addresses the need for a specific classification system for motorbike spoke wheel injuries in children, as existing systems lack the required granularity. The current gap in knowledge lies in the absence of standardized treatment protocols for these injuries, and our research aims to bridge this gap by providing a comprehensive classification system that can guide effective and consistent management strategies. The scope of this study encompasses a comprehensive analysis of SWIs in children under 14 years of age, considering both genders and various age groups. Our investigation focuses on SWIs sustained as passengers involving the foot and ankle, which were presented at a tertiary care hospital’s Accident and Emergency Department over a period of 2 years, from January 2019 to 2021. The study included children who presented with foot and ankle injuries sustained as passengers due to motorbike spoke wheels within 3 days of the incident. A primary objective of this research is to establish a new classification system specifically designed for motorbike SWIs in children. This classification system aims to categorize these injuries into distinct classes, facilitating a more precise diagnosis and enabling the development of standardized treatment protocols. By implementing this classification, we seek to improve the effectiveness and efficiency of the management of SWIs in pediatric patients. Through this study, we aim to shed light on the epidemiology, patterns, and outcomes of motorbike SWIs in children, thereby contributing to a deeper understanding of this critical public health issue. This research may serve as a valuable resource for healthcare providers, policymakers, and researchers involved in pediatric trauma care and injury prevention.

## Materials and methods

This prospective observational study was conducted in the Accident and Emergency Department of public and private sector hospitals in Karachi Pakistan from January 2019 to January 2021. All Children < 14 years of age of either gender and age with foot and ankle injury due to motorbike spoke wheels as passengers and presenting within 3 days of sustaining the injury during the study period were included. Exclusion criteria were patients presenting with bicycle injuries, foot injuries due to falls, accidents, and children of polytrauma with other associated injuries requiring surgical interventions. All the enrolled children were assessed and resuscitated as per advanced trauma life support protocol. Complete history and physical examination were done, and relevant investigations (laboratory investigations and radiographs) were ordered. The motorbike SWI was assessed for its location and classified by new classification (Table 1) by the orthopedic surgeon at the emergency department by using the classification table provided by investigators. This table provides a comprehensive overview of the classification criteria for motorbike spoke wheel injuries (SWIs) in children, aiding in the better understanding of the injury types. The new classification system for motorbike spoke wheel injuries (SWIs) in children was developed through a rigorous process involving a multidisciplinary expert panel, literature review, Delphi method, pilot testing, and validation to ensure transparency and reproducibility.

**Table 1:** Muzzammil classification of spoke wheel injury in children—Class I: Soft tissue injury without skin loss (Scratch/bruise/simple cut); Class II: Skin loss of more than 1 cm without underlying tissue involvement; Class III: Skin loss with underlying tissue involvement. This class is further divided on basis of underlying soft-tissue involvement; Class III_T_: Tendon involvement (partial tear/complete tear); Class III_B_: Bone fracture; Class III_NV_: Neurovascular involvement; Class IV: Included mangled foot/toe.

Spoke wheel injury		Management
I	Soft tissue injury without skin loss (scratch/bruise/simple cut)	Symptomatic ± antibiotics
II	Skin loss more than 1 cm without underlying tissue involvement	Debridement + antibiotic + wound coverage
III	Skin loss with underlying tissue involvement	Debridement + antibiotic + wound coverage
III_T_	Tendon involvement (partial tear/complete tear)	Surgical repair/reconstruction
III_B_	Bone fracture	Surgical fixation/casting
III_NV_	Neurovascular involvement	Surgical repair/grafting
IV	Mangled foot/toe	Amputation

After thorough wound cleansing and debridement, patients were managed according to the classification table by wound debridement, dressings, splint, skin graft, flap coverage, or combination. A non-adherent dressing was applied. Ankle immobilization with plaster of Paris slab and limb elevation was done. Antibiotics were prescribed depending on the severity of injury and culture reports.

Patients with moderate to severe injury (type II and III) were admitted in hospital after initial resuscitation and assessment by on call orthopedic surgeon in ER department, dead and necrotic tissue was debrided under anesthesia. The wound was inspected regularly, and if required, repeated debridement was performed.

In type III, underlying soft tissue damage was managed accordingly and managed by the concerned department. Flaps were added in exposed wounds, fractured bone, ruptured tendons, and/or weight-bearing areas. Ruptured tendons were repaired using Prolene 4/0, and fractures were fixed by an orthopedic surgeon. Patients were actively monitored for up to one year post-discharge, with regular follow-up appointments assessing various aspects of care and treatment effectiveness. Specific criteria were used to measure outcomes and determine the need for further interventions. This extended follow-up duration ensured a comprehensive understanding of the healing process and the impact of the study’s classification and treatment protocols. In this study, ethical approval and clearance were obtained from the Institutional Review Board (IRB). All methods were carried out in accordance with relevant guidelines and regulations. The informed consent process involved obtaining written consent from legal guardians or parents after providing a detailed explanation of the study’s objectives, procedures, with a focus on ensuring participant confidentiality and voluntary participation. Statistical analysis was done using SPSS version 25. Quantitative variables like age and time since injury were presented as mean and standard deviation. Qualitative variables like gender and side of injury were presented as frequency and percentage. Data was presented in a table where necessary. The Chi-square test was used in the statistical analysis of the given study. It was used to compare the outcome after stratification according to different variables, especially with the grade of injuries. The p-values less than 0.05 were considered statistically significant. To evaluate the inter-rater reliability of the classification system, each participating orthopedic surgeon was requested to independently apply the classification system to all patients with SWI during the study period. Once all the ratings were collected, the kappa statistic was employed to evaluate the agreement level among the raters. This statistical method enabled us to determine the degree of consistency in the classification system’s application by each surgeon.

## Results

A total of 830 children having trauma were registered in the specified duration, out of which 158 suffered SWI. The mean age was 6.2 ± 5.4 years (3–13 years). Eighty-nine (56.32%) presented at public sector hospitals while 69(43.67%) at private. Predominantly were male children127 (80.37%). All patients suffered high energy injuries (motorbike) as passengers and were sitting astride. The foot was trapped in the front wheel in 17 (10.75%) patients and the back wheel in 141 (89.24%) patients. There were 82 (51.89%) who were wearing chappal (slipper/flip flops), 62 (39.24%) were wearing shoes, and 14 (8.86%) were without footwear.

The right foot was involved in 15 (72.78%) patients and the left in 43 (27.21%). The majority (51.81, n = 82) were received within 4.2 ± 5 (1–9) hours of sustaining the injury. The peak hours of presentation were 8 to 10 AM and 12 to 2 PM and the majority (47.46%, n = 75) of children sustained these injuries on Saturday.

Forefoot was involved in 6 (3.7%) patients, midfoot in 11 (6.96%), hindfoot in 117 (74.05%), and forefoot and midfoot in (4.43%) patients, while hindfoot and the midfoot together were involved in 17 (10.75%) patients.

Class I injury was seen in 18 (11.39%) patients, class II in 69 (43.67%), and class III in 68 (43.03%) patients. Class III injury was further classified where IIIT injuries were 32 (20.25%) in which Achilles’ tendon was ruptured in 21(13.29%) children, extensor tendons in 8 (5.06%), and flexor tendons in 3 (1.8%) children. Class IIIB were 29 (18.35%) where Calcaneus was fractured in 17 (10.75%) and phalanges in 6 (3.79%) children each. Metatarsal fracture, talus fracture, medial malleolus fracture, lateral malleolus fracture, distal tibia, and distal fibula was fractured in 6 (3.79%) children each. Class IIINV injuries were identified in 7 (4.43%) in which children had posterior tibial artery and nerve transection. Class IV mangled foot/toe injuries were observed in 3 (1.8%) children (Tables 4, 5).

The flap was needed in 33 (20.88%) patients. There were no complications like flap necrosis or graft rejection. Follow-up after 1 year confirmed that all children were mobile, though some children (n = 5–0.03%) reported graft site discomfort, and some (n = 3–0.01%) had difficulty in certain movements like climbing stairs.

After stratification according to the different variables, we found no significant association of the need for flap with age, gender, the type of footwear, position, and area of foot involved. Similarly, there was no association of the grade of injury with age, gender, type of footwear, position, and area of foot involved. However, we found a significant association between the need for flap and injury grades (p = 0.0001) (Table 2).

**Table 2:** Baseline characteristics and details related to injury and its management.

	Class I (n = 18)	Class II (n = 69)	Class III_T_ (n = 32)	Class III_B_ (n = 29)	Class III_NV_ (n = 7)	Class IV (n = 3)	Total	p-value
Gender								0.028
Male	14	57	27	22	5	2	127	
Female	4	12	5	7	2	1	31	
Wheel side								0.153
Front	1	11	3	2	0	0	17	
Back	17	58	29	27	7	3	141	
Footwear								0.067
Chappal (slipper)	12	36	18	12	3	1	82	
Shoes	4	28	12	16	2	0	62	
None	2	5	2	1	2	2	14	
Side of foot								0.014
Right foot	12	50	25	19	6	3	115	
Left foot	6	19	7	10	1	0	43	
Part of foot								0.043
Forefoot	1	2	0	1	0	2	6	
Midfoot	3	7	1	0	0	0	11	
Hindfoot	10	49	28	25	5	0	117	
Forefoot and midfoot	1	4	0	1	0	1	7	
Midfoot and hind foot	3	7	3	2	2	0	17	
Treatment needed								0.021
Flap needed	0	13	10	6	4	0	33	
Skin graft needed	0	26	11	9	2	1	49	

As shown in Table 3, it is clear how there is a difference between the widely accepted Tscherne and Oestern classification and our proposed classification. Muzammil’s classification further classify SWIs associated with underlying tissue damage including bone, tendon, and neurovascular structure, which gives a more simplified method to communicate and manage according to the type of injury. The kappa value for our observations was 0.8 reflecting that majority of orthopedic surgeons experienced no difficulty in application of this classification.

**Table 3:** Classification of the spoke wheel injuries according to the older as well as our new classification.

Muzzammil classification of spoke wheel injury in children			Tscherne and Oestern classification			p-value
Class I	Soft tissue injury without skin loss (scratch/bruise/simple cut)	18 (11.39%)	Grade I	Minor bruises and lacerations	18 (11.39%)	0.988
Class II	Skin loss more than 1 cm without underlying tissue involvement	69 (43.67%)	Grade II	Major soft tissue loss	69 (43.67%)	0.988
Class III	Skin loss with underlying tissue involvement		Grade III	Tendon rupture, neurovascular injury, and fractures	71 (68.1%)	0.001
Class III_T_	Tendon involvement (partial tear/complete tear)	32 (20.25%)				
Class III_B_	Bone fracture	29 (18.35%)				
Class III_NV_	Neurovascular involvement	7 (4.43%)				
Class IV	Mangled foot/toe	3 (1.8%)				

## Discussion

In the last decade, there has been a drastic increase (400%) of motorcycles in Pakistan^19^. This study showed an alarming trend of children presenting with SWI while riding on a motorbike. The reason for such a high incidence in our setup is no age limitation on riding, lack of wheel guards and footrests, and overloading. Motorbike spokes produce a more severe injury to the foot and ankle because of their higher speed and energy.

When we searched the literature we found that SWIs due to bicycles and motorbikes were studied together by previous authors^10,11^, but Zhu et al.^16^ believed that motorbike SWIs had different mechanisms and severity than those of a bicycle and must be studied separately. We agree with Zhu et al. and conducted this study on the pediatric motorbike SWIs. They proposed a four-grade treatment-based classification for motorbike SWIs in their study. In grade I, the Achilles tendon was exposed but not cut and was treated with flap transfer. In grade II, the ruptured Achilles tendon was repaired and coverage was achieved with flap. In grade III, injuries associated with calcaneus fractures were stabilized. In grade IV, the mangled foot needed amputation^16^. This classification only applied to SWIs requiring surgical intervention including tendon repair and flap coverage, minor injuries and vascular involvement were not included in the study.

In the study of Segers et al.^20^, Mine et al.^21^, and Suri et al.^22^, the minor lacerations of the heel skin were taken as one group. Our experience showed that these minor injuries, which were very common in a bicycle spoke injuries, were rarely encountered in the motorbike spoke injuries. Therefore, Minor injuries are classified in class I in our classification rather than taken as a group.

Suri et al.^22^ assessed the record of 42 children with a mean age of 14.9 years. They used their classification and divided the SWIs into three classes. Class I injuries included avulsion of minor degree noted in seven children. Class II injuries were avulsion of the heel which were extensive but without exposure of tendons and bones and documented in 17 children. Class III injuries involved avulsion of the heel with exposed tendons, bones, and vessel damage present in 18 children^22^. Suri found his classification best suited for the Indian population, but critics believe that this classification had not included fractures that can occur with SWIs^23^. The fractures, vascular involvement, and amputation play important roles in our classification system, compared with the previous grading systems, this classification system seemed to be simpler for implementation, covering almost all structure involvement and more special for motorbike spoke injuries. We have compared our classification with the grades of injury given by Das De and Pho^9^ Mine et al.^21^, Suri et al.^22^, those patients with fractures were not included by them and Zhu et al. grading system is only for heel injuries and did not include underlying vascular injuries (Tables 4, 5)^16^.

**Table 4:** Summarizing the different classification systems used for spoke wheel injuries in the literature.

Study	Classification system	Grades	Included injuries
Das De and Pho^9^	Simple	Not specified	Not specified
Mine et al.^21^	Modified Eichenholtz	Not specified	Minor lacerations, no fractures
Suri et al.^22^	Indian classification	Class I: minor avulsion; Class II: extensive avulsion without tendon or bone exposure; Class III: avulsion with tendon, bone, and vascular damage	Minor lacerations, no fractures
Zhu et al.^16^	Motorbike SWI classification	Grade I: exposed but not cut Achilles tendon; Grade II: ruptured Achilles tendon with flap coverage; Grade III: calcaneus fractures stabilized; Grade IV: mangled foot requiring amputation	Not specified
Present study	Muzzammil classification for spoke wheel injuries in children	Class I: minor injuries involving only skin; Class II: fractures and soft tissue injuries without tendon, bone, or vascular involvement; Class III: fractures, tendon or vascular involvement; Class IV: amputation required	Fractures, tendon and vascular injuries

**Table 5:** Fracture, tendon injuries and their managements.

Injury classification	Number of patients	Subcategories	Treatment
Class I	18 (11.39%)		
Class II	69 (43.67%)		
Class III	68 (43.03%)	IIIT (32), IIIB (29), IIINV (7)	
IIIT	32 (20.25%)	Achilles’ tendon rupture (21)	Surgical repair, casting, rehabilitation
		Extensor tendon injuries (8)	
		Flexor tendon injuries (3)	
IIIB	29 (18.35%)	Calcaneus fracture (17), phalanges fracture (6)	Surgical repair, casting, rehabilitation
		Metatarsal fracture (6), talus fracture (6)	
		Medial malleolus fracture (6)	
		Lateral malleolus fracture (6)	
		Distal tibia fracture (6)	
		Distal fibula fracture (6)	
IIINV	7 (4.43%)	Posterior tibial artery and nerve transection	Surgical repair, vascular intervention
			Nerve repair, rehabilitation
Class IV	3 (1.8%)	Mangled foot/toe injuries	Amputation, rehabilitation

In our study, only 11.39% of children presented with class I injury, classified and managed in the accident and emergency department by an orthopedic surgeon. Higher the grade, the likelihood of multiple debridements increased. We noted that treatment and prognosis were directly related to the grade of injury, with a direct decision of the treatment at the time of classification, especially that of the fracture, tendon involvement, and neurovascular damage, which was not possible with the older classification. Our classification is highly specific for SWI, with grades exactly based on the number of structures involved.

We admitted Class II and III injured patients after initial debridement after initial assessment in the emergency department. Re-evaluation of all patients done in 48 h. No wound was primarily stitched on the day of admission as vascularity is compromised^23^. Tendons were repaired, Graft and flap were performed and fractures stabilized if needed.

We recommend a thorough local assessment of all children with motorbike SWIs. They can sometimes deceptively appear as minor injuries and, hence, not be managed properly. Therefore, a high level of suspicion should be used when dealing with all SWIs admitting those cases that fall in the moderate and severe categories. Strict legislation regarding the proper use of the motorbike, the age restriction for child passengers, and wearing protective shoes should be mandatory. Improvement in the design of the motorbike with adequate spoke guards or shields on both sides and an adjustable footrest is required. This can decrease the frequency and severity of motorbike SWIs in our country. The limitations of this study are that we did not calculate healing time, and although we followed up, a detailed psychological or motor examination was not undertaken.

### Limitations of the study

#### Small sample size

The study suffered from a limited number of participants due to its single-hospital setting, impacting the generalizability of results.

#### Single-hospital setting

Focusing on one hospital only may not fully represent the broader population or regional variations, highlighting the need for multi-center studies.

### Areas for future research

#### Multicenter studies

Future research should involve multiple healthcare facilities to confirm the proposed classification system’s validity and applicability in diverse settings.

#### Longitudinal studies

Evaluating the new classification system’s long-term effectiveness in guiding treatment decisions and improving patient outcomes over time is essential.

#### Comparative analyses

Comparative studies between the proposed classification and existing systems should be conducted, considering different epidemiological profiles of SWIs.

#### Public health interventions

Research should focus on evaluating the effectiveness of public health interventions, such as legislative measures and safety awareness campaigns, to prevent motorbike SWIs in children.

## Conclusion

Here in this current study, a new classification system specific for a motorbike SWIs has been introduced along with its application on children presenting at tertiary care hospital’s emergency department. It has been observed that there is a rising prevalence of these entrapment injuries. Application of the proposed classification will make universal management guidelines for these SWIs, especially in the Indo-Pak region where these are very common.

## Data Availability

The datasets used and/or analyzed during the current study are available from the corresponding author on reasonable request.
